# PyElph - a software tool for gel images analysis and phylogenetics

**DOI:** 10.1186/1471-2105-13-9

**Published:** 2012-01-13

**Authors:** Ana Brânduşa Pavel, Cristian Ioan Vasile

**Affiliations:** 1 Department of Automatic Control and Systems Engineering, Politehnica University of Bucharest, Splaiul Independenţei, No. 313, Bucharest, Romania

## Abstract

**Background:**

This paper presents PyElph, a software tool which automatically extracts data from gel images, computes the molecular weights of the analyzed molecules or fragments, compares DNA patterns which result from experiments with molecular genetic markers and, also, generates phylogenetic trees computed by five clustering methods, using the information extracted from the analyzed gel image. The software can be successfully used for population genetics, phylogenetics, taxonomic studies and other applications which require gel image analysis. Researchers and students working in molecular biology and genetics would benefit greatly from the proposed software because it is free, open source, easy to use, has a friendly Graphical User Interface and does not depend on specific image acquisition devices like other commercial programs with similar functionalities do.

**Results:**

PyElph software tool is entirely implemented in Python which is a very popular programming language among the bioinformatics community. It provides a very friendly Graphical User Interface which was designed in six steps that gradually lead to the results. The user is guided through the following steps: image loading and preparation, lane detection, band detection, molecular weights computation based on a molecular weight marker, band matching and finally, the computation and visualization of phylogenetic trees. A strong point of the software is the visualization component for the processed data. The Graphical User Interface provides operations for image manipulation and highlights lanes, bands and band matching in the analyzed gel image. All the data and images generated in each step can be saved. The software has been tested on several DNA patterns obtained from experiments with different genetic markers. Examples of genetic markers which can be analyzed using PyElph are RFLP (Restriction Fragment Length Polymorphism), AFLP (Amplified Fragment Length Polymorphism), RAPD (Random Amplification of Polymorphic DNA) and STR (Short Tandem Repeat). The similarity between the DNA sequences is computed and used to generate phylogenetic trees which are very useful for population genetics studies and taxonomic classification.

**Conclusions:**

PyElph decreases the effort and time spent processing data from gel images by providing an automatic step-by-step gel image analysis system with a friendly Graphical User Interface. The proposed free software tool is suitable for researchers and students which do not have access to expensive commercial software and image acquisition devices.

## Background

PyElph is an open source Python based software for gel images analysis which can be used for different molecular biology or genetics studies. The software is able to analyze genetic variations of the DNA molecules from different species or populations. PyElph analyses gel image patterns of DNA genetic markers and generates phylogenetic trees based on the information available in a gel image. Thus, the software can be successfully used for population genetics, phylogenetics and taxonomic studies. An important feature of PyElph is its interactive Graphical User Interface (GUI) which has a simple design that makes the program easy to use and learn.

Genetic variation can be studied using molecular techniques based on genetic markers. DNA fragments obtained through these techniques are used to estimate the similarity between samples of DNA sequences. Genetic markers are used for various studies and genetic tests, such as paternity tests, forensic tests, studies of intra- and inter- population polymorphisms [1,2], taxonomic classifications, genetic mapping. A few examples of genetic markers used for phylogenetic and genomic studies are RFLP (Restriction Fragment Length Polymorphism), AFLP (Amplified Fragment Length Polymorphism), RAPD (Random Amplification of Polymorphic DNA), STR (Short Tandem Repeat). Genetic markers based techniques usually use PCR (Polymerase Chain Reaction) to amplify the DNA fragments and gel electrophoresis to separate them. After migration (separation) of the samples in the electrophoresis gel, a photo of the resulting pattern is taken by a common digital camera or a dedicated system. PyElph automatically detects the migration lanes and bands, computes the molecular weight of each separated fragment, matches the bands from all samples, based on their migration distance, computes similarity and distance matrices which are then used to generate phylogenetic trees.

The authors are aware of other software systems for gel images analysis which have similar functionalities to PyElph. Such a software tool is the commercial program QuantityOne from Bio-Rad, which offers automatic lane/band detection, band matching, molecular weight computation and phylogenetic tree computation and display. On the other hand QuantityOne includes additional features (colony counting, data acquisition from Bio-Rad devices, etc.), but it is very expensive and has a complex design, which requires prior training of the user. In contrast, PyElph is free and easy to learn and use, because it is oriented on gel image analysis applications and has a friendly GUI. Moreover, PyElph is a general tool that can be used with simple gel image acquisition systems (for example, a UV box with a camera on tripod). Another example of a gel image analysis software is the java based program GelAnalyzer, freely available at http://www.gelanalyzer.com. However, this software does not include phylogenetic analysis and is not open source. A big advantage of PyElph is that new functionalities can be added by users according to their needs. Thus, science laboratory courses and low budget research laboratories would benefit greatly from the proposed software.

## Implementation

**Figure 1:**
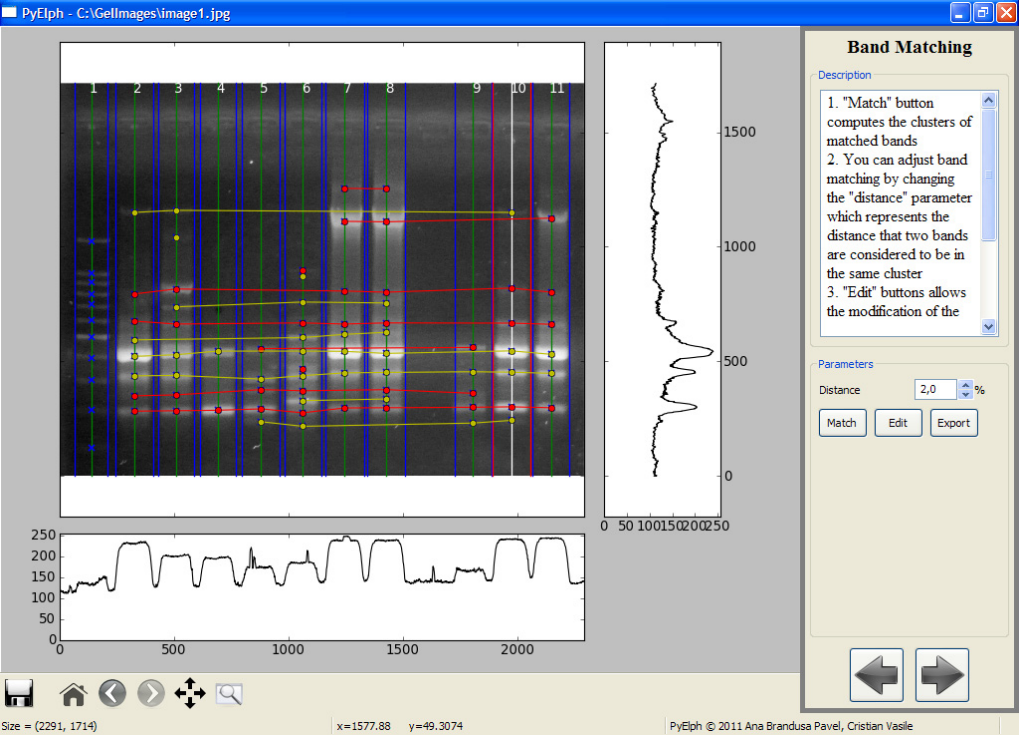
**PyElph GUI - the band matching step**. The figure is a screen capture of PyElph band matching step. Lanes, bands and band matching can be observed in the figure, as well as some GUI components, the toolbar, the options panel for the current step and the image view.

PyElph GUI is structured in six processing steps and has three main components: a toolbar, an options panel and the image view (Figure 1). The operations for manipulating and editing the image are embedded in toolbar. The options panel contains the parameters and important operations of each processing step. And finally the image view displays the processed image or the phylogenetic trees.

The functionalities provided by the proposed software system are structured in six steps, as follows:

**Step 1 **The program opens the first window of the GUI which provides the following operations, available from the toolbar:

• *Load an image *- PyElph accepts the following formats: gif, png, jpg, tiff, bmp. The image can be either grayscale or RGB, but it is automatically converted to grayscale;

• *Editing operations *: crop, rotate;

• *Navigation operations *(which are available for all the 6 steps): *zoom in*, *zoom out*, *move the view*, *back*, *forward*, *home*;

• *Save*, which is also available for all the 6 steps;

For the next processing steps, the image must be placed such that the wells are situated at the top of the image view. After the image was prepared, the button *next*, placed on the option panel, will lead to the next step.

**Step 2 **is responsible for lane detection. Lanes are automatically detected by accessing the *detect *button, but the software also provides manual detection or removal of the lanes and manual adjustment of the detection. If the image has a low quality and the automatic detection fails, it is recommended to use the option (*define lane width*) which allows the operator to graphically define the lane's width and improve the automatic lane detection. The method implemented for automatic lane detection is based on computing the maximum value of each pixels column (which is shown in the graph situated below the image view in Figure 1). The mean value of the lanes' width is computed by using a threshold, set to 70% of the maximum value of the domain. By considering this threshold with a high intensity value, a lot of noise is eliminated and the probability that the detected lanes are real lanes is higher. Using the lanes detected at this threshold level, a mean value for the lanes' width is computed and the lanes thinner than the mean width are eliminated. Then the mean width of the selected lanes is again computed and it is used to detect all the lanes, between two thresholds (70% and 15%), which have a width deviation of 25% from the mean width value. The width deviation value can be modified from the GUI. After the lanes are detected, the operation of background subtraction is performed. The GUI also provides an option to visualize the image after background subtraction. After lane detection is finished, the next step can be accessed.

**Step 3 **is dedicated to band detection. At this point all the bands that correspond to real molecular fragments are detected and marked with an **x**. Band detection is made automatically, but it can also be adjusted by changing some parameters or by manually adding or removing bands. Automatic band detection is performed by summing the columns of a lane and comparing the values obtained with an intensity threshold set to 20 multiplied with the lane's width. This threshold can be modified from the GUI. To avoid the detection of false peaks, a moving average filter was applied on the data. The width of the filter and the filter passes can be modified from the GUI to improve detection. The filter width determines how many values are used to compute the average for the current position as follows: $x_{F}\left[i\right]=\frac{1}{2*width+1}\cdot \sum_{k=i-width}^{i+width}x\left[k\right]$. The passes filter parameter represents the number of times the filter is applied on the data. For large images it is recommended to use higher values for both the filter width and the filter passes. By selecting a lane, the graph on the right side of the image view shows the values of the pixels corresponding to the column situated in the middle of a lane (lane 10 in Figure 1). So, each peak corresponds to a band in the selected lane. After band detection is finished, the next step can be accessed.

**Step 4 **is optional and provides molecular weights computation using a defined molecular weight marker. The lanes which contain molecular markers must be selected from the GUI. Then, one marker is used to compute all the molecular weights, based on a defined standard. New standards can be easily defined by introducing the number of bands and then, the corresponding molecular weights in a decreasing order. The new defined standard is a text file with .*marker *extension and it is stored in a local directory (*standards*). After it is defined, the new standard is saved automatically and it will be available in the interface for future use. The molecular weights are fitted after a linear electrophoresis migration model:

$$\text{ln}\left(Weight\right)=\alpha *Migration\;Distance+\beta ,\alpha <0,\beta >0$$

The parameter *α *of the model is negative because the migration distance of the fragments is inversely proportional to the logarithm of their molecular weights. *β *is a positive offset due to the distance from the top of the image to the wells.

**Step 5 **provides the operation of band mathing (clustering) as shown in Figure 1. The clusters are represented as horizontal lines or isolated points (in case a cluster contains only one band). The clusters are also colored by alternating red and yellow, such that they can easily be differentiated. A heuristic algorithm for band mathing was developed such that any two bands in a cluster satisfy two conditions: they don't belong to the same lane and the distance between them is less than 2% of the image's height (this value can be modified from the GUI). Thus, a clustering matrix is obtained which can be modified and saved from the GUI. Each column of the clustering matrix is a sample (population, species) and each line represents a cluster. The presence or absence of a band is marked with 1 (or +), respectively 0 (or -). This matrix is used to compute the similarity matrix using Dice coefficient [3] which expresses the similarity level between two DNA patterns. For samples *S_i _*and *S_j _*the similarity is:

$$Dice\left(S_{i},S_{j}\right)=\frac{2*number\;of\;common\;bands\;in\;lanes\;i\;and\;j}{number\;bands\;in\;lane\;i\;+\;number\;bands\;in\;lane\;j}\left[\%\right]$$

Then, the distance matrix is computed as 100 times matrix of ones minus the similarity matrix. This matrix is used for generating phylogenetic trees by clustering methods.

**Step 6 **computes phylogenetic trees based on clustering methods applied on the distance matrix computed in the previous step: Neighbor Joining [4,5], UPGMA (Unweighted Pair Group Method with Arithmetic Mean), WPGMA (Weighted Pair Group Method with Arithmetic Mean), Single Linkage, Complete Linkage [6]. The method is selected from the GUI and then the corresponding tree can be displayed. The labels of the analyzed samples can be modified from the GUI and the genetic distances can be displayed on the branches of the tree.

**Figure 2:**
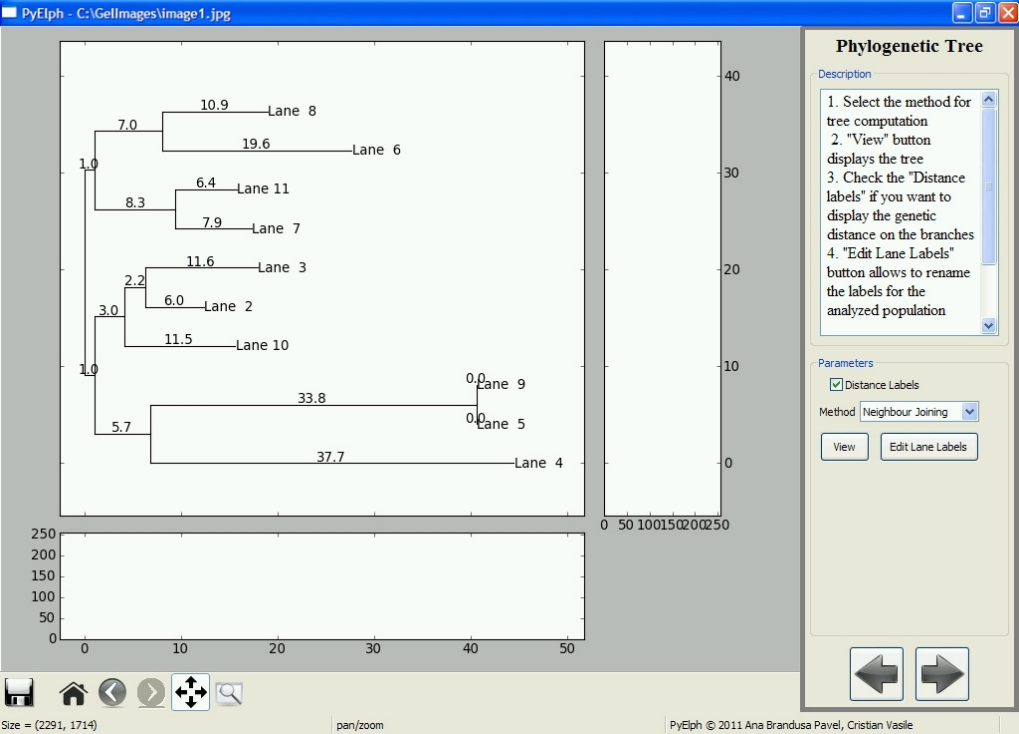
**PyElph GUI - the phylogenetic tree step**. The figure is a screen capture of PyElph phylogenetic tree step. The figure shows a phylogenetic tree computed using Neighbor Joining method and data from figure 1.

An example of a phylogenetic tree generated by PyElph using Neighbor Joining method is provided in Figure 2. The tree was computed based on the information extracted from the gel image shown in Figure 1. The genetic distances are also displayed on the branches. The distance matrix corresponding to the gel image in Figure 1 was computed and saved using PyElph. In order to verify the phylogenetic tree computation, the distance matrix was introduced in the online Phylip package for phylogeny inference, in the required format (http://mobyle.pasteur.fr/cgi-bin/portal.py#forms::neighbor). The phylogenetic tree generated by Phylip was compared to the one obtained using PyElph. The results correspond and the lengths of corresponding branches of the two trees have equal values.

The video clip (additional file 1) shows PyElph's functionalities and options and is a step-by step demon- stration of how to use the software tool.

## Results and Discussion

PyElph has been tested on over 50 agarose gel images in which DNA molecules were stained with ethidium bromide which is a fluorescent dye and the pictures were taken using an UV transilluminator and a common digital camera. Patterns of different genetic markers: Random Amplification of Polymorphic DNA (PCR-RAPD) [7], Amplified Ribosomal DNA Restriction Analysis (ARDRA) which is an extension of RFLP technique [8] and Repetitive Extragenic Palindrome (REP-PCR) [9] have been analyzed for population genetics studies and taxonomic classification of new isolated bacteria.

As mentioned in the Background section, PyElph provides similar functionalities, regarding gel images analysis, with the commercial program Quantity One from Bio-Rad. Other programs for gel image processing are GelAnalyzer, which is freely available, but not open source, and the software package proposed in [10], which provides automatic allele classification. The main advantages of PyElph towards other gel images analyzing systems are the following:

• it is free and open source;

• it provides a very easy to use GUI which allows visualization of the processed data in a suggestive and interactive manner;

• it is implemented in Python, so it can be integrated in bigger bioinformatics packages, like Biopython for example; it can also be extended by the bioinformatics research community;

• it runs on both Windows and Linux platforms;

• it provides both molecular weights and phylogenetic trees computation and visualization;

• it implements a band matching algorithm;

• it provides access to different parameters which contribute to automatic detection adjustments;

• it is independent of the gel image acquisition device and it can be used with simple, low cost systems.

PyElph can be extended and reused very easily. Future work includes combining data from multiple gel images to infer phylogenetic trees. It is of current research to determine how to combine data obtained from different experiments of the same samples (for example by using different primers in the PCR-RAPD technique) in order to compute trees which describe phylogenetic relations better. The interface and methods of PyElph will also be reused to build tools for microarray analysis and colony counting.

## Conclusions

This paper proposes PyElph, a free and open source software, implemented in Python, which analysis gel images and generates phylogenetic trees. The methods used for its implementation have been presented. The originality of the software tool is its GUI design, based on six steps that gradually lead to the results, and also, the clustering algorithm implemented for the band matching step. The program is very easy to use due to its expressive and interactive Python based GUI. It is an useful tool which has been tested on real molecular data. Thus, PyElph can be used by researchers and students who work in the fields of molecular biology and genetics.

## Availability and Requirements

• Project name: PyElph

• Project home page: http://sourceforge.net/projects/pyelph/files/releases/

• Operating system(s): Platform independent

• Programming language: Python 2.x

• Other requirements: Python 2.5 or higher, Numpy, PIL (Python Image Library), wxPython, matplotlib

• License: GNU GPL v3

• Any restrictions to use by non-academics: restrictions specified by GNU GPL v3

Before the application can be started, the following open source Python packages must be installed:

1. Python 2.5 or 2.6 interpreter - http://python.org/download/releases/

2. The following Python packages for Python 2.5 or 2.6:

(a) Numpy - http://numpy.scipy.org or http://sourceforge.net/projects/numpy/files/NumPy/

(b) PIL - http://www.pythonware.com/products/pil/

(c) wxPython - http://www.wxpython.org or http://sourceforge.net/projects/wxpython/files/wxPython/

(d) Matplotlib - http://matplotlib.sourceforge.net or http://sourceforge.net/projects/matplotlib/files/matplotlib/matplotlib-1.0/

After instalation of the required packages is ready, PyElph can be started by double-clicking on *PyElph.py *python script. The folder *standards *contained the defined weight marker standards. New weight marker stadards can be defined and saved from PyElph GUI or by editing a text file with the .*marker *extension.

For the convenience of Windows users, an installation kit is also available at http://sourceforge.net/projects/pyelph/files/releases/PyElph1.3-installer.exe/download. The kit installs a windows executable version of PyElph, which was generated with *py2exe*. Therefore, there is no need to install Python or other python packages to run this executable version of PyElph.

## Authors' contributions

Both authors contributed to the development of all parts of PyElph. ABP provided the biological expertise and had a significant contribution to the back-work of the program: lane and band detection, weight computation, band matching and phylogenetic tree generation. She also tested all the components and ensured that the program runs smoothly and correct. CIV was involved with the GUI design and implementation. He integrated matplotlib with wxPython in order to enhance the interaction with the user. He also implemented the display method for the phylogenetic trees. Both authors developed the clustering algorithm that is used for band matching. All authors read and approved the final manuscript.

## Supplementary Material

Additional file 1 **PyElph demo video clip**. The *PyElphDemo.mp4 *mpeg-4 video presents most of the features of PyElph. It follows all six processing steps. Click here for file
